# Significant variability exists in the cytotoxicity of global methicillin-resistant *Staphylococcus aureus* lineages

**DOI:** 10.1099/mic.0.001119

**Published:** 2021-12-20

**Authors:** Maisem Laabei, Sharon J. Peacock, Beth Blane, Sarah L. Baines, Benjamin P. Howden, Timothy P. Stinear, Ruth C. Massey

**Affiliations:** ^1^​ Department of Biology and Biochemistry, University of Bath, Bath, BA2 7AY, UK; ^2^​ Department of Medicine, Addenbrooke’s Hospital, University of Cambridge, Hills Road, Box 157, Cambridge, CB2 0QQ, UK; ^3^​ Department of Microbiology and Immunology, University of Melbourne at the Peter Doherty Institute for Infection and Immunity, Melbourne, Australia; ^4^​ Microbiological Diagnostic Unit Public Health Laboratory, University of Melbourne at the Peter Doherty Institute for Infection and Immunity, Melbourne, Australia; ^5^​ School of Cellular and Molecular Medicine, University of Bristol, Bristol, BS8 1TD, UK

**Keywords:** *Staphylococcus aureus*, cytotoxicity, bacterial pathogenesis, bacterial virulence

## Abstract

*
Staphylococcus aureus* is a major human pathogen where the emergence of antibiotic resistant lineages, such as methicillin-resistant *S. aureus* (MRSA), is a major health concern. While some MRSA lineages are restricted to the healthcare setting, the epidemiology of MRSA is changing globally, with the rise of specific lineages causing disease in healthy people in the community. In the past two decades, community-associated MRSA (CA-MRSA) has emerged as a clinically important and virulent pathogen associated with serious skin and soft-tissue infections (SSTI). These infections are primarily cytotoxin driven, leading to the suggestion that hypervirulent lineages/multi-locus sequence types (STs) exist. To examine this, we compared the cytotoxicity of 475 MRSA isolates representing five major MRSA STs (ST22, ST93, ST8, ST239 and ST36) by employing a monocyte-macrophage THP-1 cell line as a surrogate for measuring gross cytotoxicity. We demonstrate that while certain MRSA STs contain highly toxic isolates, there is such variability within lineages to suggest that this aspect of virulence should not be inferred from the genotype of any given isolate. Furthermore, by interrogating the accessory gene regulator (Agr) sequences in this collection we identified several Agr mutations that were associated with reduced cytotoxicity. Interestingly, the majority of isolates that were attenuated in cytotoxin production contained no mutations in the *agr* locus, indicating a role of other undefined genes in *S. aureus* toxin regulation.

## Introduction

*Staphylococcus aureus* is responsible for a wide array of diseases ranging from superficial skin infections to severe, life-threatening cases of pneumonia and bacteraemia [1]. The emergence of antibiotic resistant lineages including methicillin-resistant *S. aureus* (MRSA) has complicated treatment. For decades the circulating MRSA lineages appeared to be limited to causing infections within healthcare settings in patients with predisposing conditions who were susceptible to infection [2]. However, in the 1990s distinct MRSA lineages, unrelated to earlier circulating MRSA lineages, started to emerge outside of healthcare settings to cause infections in otherwise healthy individuals [2, 3]. Community-associated MRSA (CA-MRSA) isolates result in similar clinical manifestations, such as severe skin and soft-tissue infections (SSTIs), despite broad genetic diversity among CA-MRSA lineages [4]. Understanding the differences between the healthcare restricted MRSA lineages and the more recently emerged CA-MRSA lineages has been the focus of much attention [3].

Molecular and epidemiological studies of CA-MRSA isolates have identified multiple putative virulence factors, namely toxins, associated with the hypervirulent phenotype characteristic of CA-MRSA isolates. The majority of CA-MRSA isolates harbour *lukSF-PV* which encodes the bi-component pore-forming leucocidin [4]. Human neutrophils have been shown to be highly susceptible to PVL-mediated lysis due to the expression of complement receptors, C5aR and C5L2, which are required for PVL binding [5]. Importantly, the role of PVL in pathogenesis is likely dependent on the site of infection; a clear role is observed in a rabbit necrotizing pneumonia model [6], but the role of this toxin in dermonecrosis is less evident [7, 8]. Aside from PVL, over-expression of core genome virulence determinants, notably α-haemolysin (Hla) and α-type phenol-soluble modulins (α-PSMs), has been hypothesised to significantly contribute to the enhanced virulence of CA-MRSA isolates [9–12]. Hla is the prototypical β-barrel pore-forming cytotoxin [13]. Multiple studies utilising serological and animal models of infection have indicated a prominent role for Hla in the pathogenesis of disease [14]. The α-PSMs are characterised as small amphipathic α-helical peptides that efficiently lyse numerous cell types, independent of cell specific receptor [11, 15]. As with Hla, α-PSMs significantly contribute to virulence in a murine infection models of bacteraemia and skin lesions [11].

Toxin regulation in *S. aureus* is governed primarily by the accessory gene regulator (*agr*), which employs a cell density dependent, quorum sensing system to upregulate a suite of secreted virulence factors such as toxins and downregulate surface binding proteins [16]. Accordingly, the *agr* system plays a central role in the development of a range of *S. aureus* infections, most notably in CA-MRSA skin infections [17]. Furthermore, transcriptomic data indicate enhanced *agr* regulation of important toxins (PVL, Hla and α-PSM), which are frequently associated with CA-MRSA virulence [17]. Together, these studies have contributed to the frequent use of the term ‘hypervirulent’ when referring to CA-MRSA lineages.

Recently, studies of toxicity at a population level have reported that this phenotype varies widely within individual MRSA multi-locus sequence types (STs) [18], suggesting that the virulence of an individual MRSA isolate should not be inferred from its genotype [18]. Non-cytolytic clinical isolates are commonly referred to as ‘Agr dysfunctional’, due to the frequency at which mutations occur within the sensor kinase (AgrC) or response regulator (AgrA) encoding genes, impacting significantly on toxin expression. To examine this in greater detail, and across a globally representative collection of isolates, we focussed on a collection of 475 MRSA isolates representing five major MRSA STs (ST22, ST93, ST8, ST239 and ST36; Table S1, available in the online version of this article for further details) collected from Europe, North and South America, Asia and Australia, and examined their toxicity based on their ability to lyse human cells. As no single cell line exists that is susceptible to all of the toxins secreted by *S. aureus *, we use the THP-1 monocyte-macrophage cell line, which based on our empirical evidence, is susceptible to the widest range of *S. aureus* toxins, expressing receptors for PVL [5] and Hla [19] and susceptible to δ-haemolysin and α-PSMs [20]. Our findings indicate that toxicity varies significantly within STs and therefore lineage should not be used as a metric to infer pathogenicity. In addition, we have identified novel Agr mutations associated with attenuated toxicity and confirm that multiple isolates with reduced cytotoxicity have no mutations within the Agr operon, indicating the existence of other undefined toxin regulating genes in *S. aureus *.

## Methods

### Bacterial isolates and growth conditions

A list of the MRSA clinical isolates used in this study can be found in Table S1. *S. aureus* isolates were grown overnight in 5 ml of Tryptic-Soy Broth (TSB; Sigma) in a 30 ml glass tube at 37 °C with shaking at 180 r.p.m. Overnight cultures were used to inoculate 5 ml of fresh TSB at a dilution of 1 : 1000 and incubated for 18 h at 37 °C with shaking at 180 r.p.m. Under these growth conditions all *S. aureus* clinical isolates reached an OD_600nm_ within the range of 4.5–5.5 after 18 h growth. *S aureus* toxin containing supernatants were harvested from 18 h cultures by centrifugation at 14600 r.p.m. for 10 min. All clinical isolates have been genome-sequenced as described previously [18, 20–24]. Paired-end reads for these isolates were mapped to the following reference isolates: ST22 (HO 50960412 [24]), ST93 (JKD6159 [21, 22]), ST8 (USA300 strain LAC [20]), ST239 (TW20 [18, 23]) and ST36 (MRSA252 [24]). Data have been deposited in the European Nucleotide Archive under the following accession numbers ST239 (ERP000228 and ERA000102), ST22 and ST36 (ERP000871), ST93 (SRA026511.1) and ST8 (PRJEB2870).

### THP-1 cell culture

THP-1 monocyte-macrophage cell line (ATCC#TIB-202) was routinely grown in suspension in 30 ml of RPMI-1640 medium (Gibco: 11340892), supplemented with 10 % heat-inactivated foetal bovine serum (FBS) (Sigma: F7524), 1 µM l-glutamine, 200 units ml^−1^ penicillin and 0.1 mg ml^−1^ streptomycin (Sigma: G6784) at 37 °C in a humidified incubator with 5 % CO_2_. THP-1 cells were routinely viewed microscopically and sub-cultured every 3–4 days. For use in cytotoxicity assays, cells were collected by centrifugation at 1000 r.p.m. for 5 min at room temperature and resuspended to a final density of 1–1.2×10^6^ cells ml^−1^ in tissue grade phosphate buffered saline (Gibco). Cell viability was analysed using the Guava ViaCount reagent (Luminex) and easyCyte flow cytometry, typically yielding >95 % viability following THP-1 collection.

### Cytotoxicity assay

The cytotoxicity assay was optimised previously [20]. Briefly, to evaluate *S. aureus* toxicity, 20 µl of harvested supernatant (either used as 100 % or diluted to 70%, 30 % or 10 % in TSB) was incubated with 20 µl of washed THP-1 cells for 12 min at 37 °C under static conditions. Cell death was quantified using the Guava ViaCount reagent and easyCyte flow cytometry according to manufacturer’s instructions. The toxicity of each isolate was measured with two technical repeats and three biological repeats with error bars indicating the standard deviation (SD). LAC (a highly toxic CA-MRSA isolate) supernatant and TS broth were used as positive and negative controls, respectively.

### Statistical analysis

A one-way ANOVA with Tukey’s multiple comparison test was used to examine differences between experimental results (GraphPad Prism v9.0), where a *P* value<0.05 was considered to be statistically significant.

## Results and discussion

### *S. aureus* cytotoxicity is highly variable both between and within sequence types

**Fig. 1.**
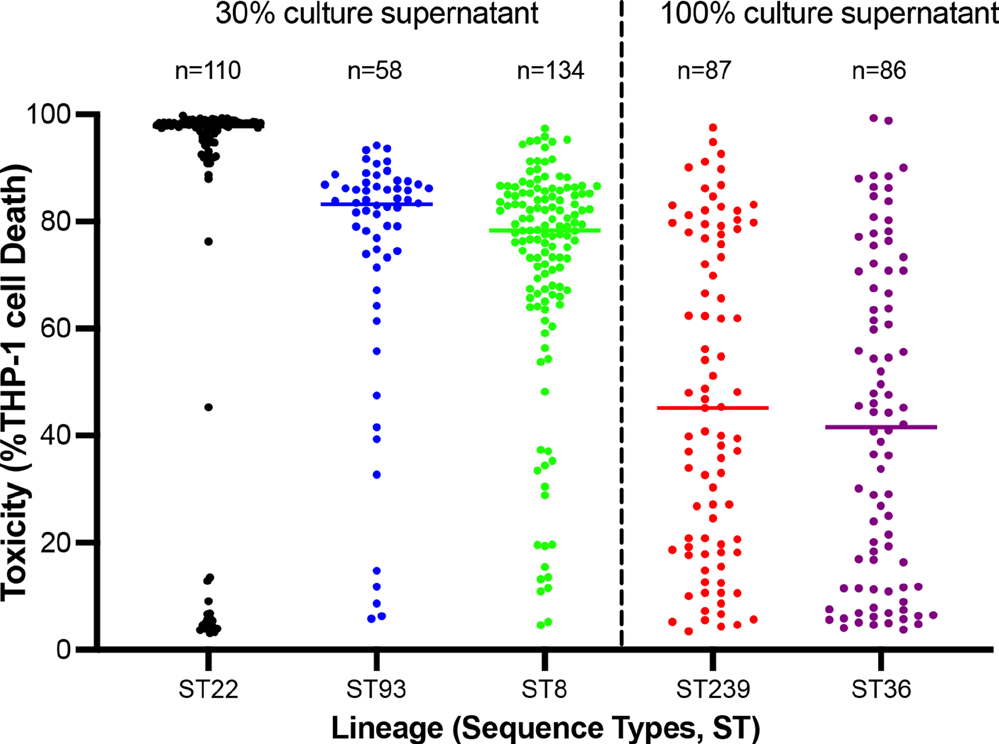
Variation in cytotoxicity between and within MRSA sequence types. The cytotoxicity of each isolate from five MRSA STs (ST22 (*n*=110), ST93 (*n*=58), ST8 (*n*=134), ST239 (*n*=87) and ST36 (*n*=86)) was quantified by incubating cell-free supernatant (either diluted to 30 % using sterile TS broth or used undiluted (100%)) with cultured THP-1 cells and cytotoxicity examined by flow cytometry. The cytotoxicity of each isolate was quantified using three biological repeats with a single dot representing the mean value for each isolate and the median of each sequence type indicated by the horizontal bars.

In this study we compared the cytotoxicity profiles of previously published collections of MRSA isolates (i.e. sequence type (ST) 8 [20], ST22 and ST36 [24]; *n*=330) with newly derived toxicity profiles (ST93 and ST239; *n*=145) to provide a comprehensive and globally distributed picture of toxin expression by *S. aureus *. What was immediately apparent was that the individual STs contained numerous isolates that were either extremely toxic (100 % cell death) or non-toxic (0 % cell death), and could not be reliably assayed under the same conditions. As a result, we used the supernatant of two of the STs (ST239 and ST36) undiluted (100%) and for the other three STs (ST22, ST93 and ST8) we diluted them to 30 % (vol/vol) in TSB (Fig. 1). The cytotoxicity of each isolate was measured using three biological repeats with high reproducibility (Fig. S1). The proportion of ST239 and ST36 isolates that killed more than 50 % of the THP-1 cells was 44 and 39 %, respectively. By contrast, the proportion of ST8, ST22 and ST93 isolates that killed more than 50 % of cells was 87, 84 and 85 %, respectively. Given both the difference in the proportion of isolates killing more than 50 % of the cells and the differences in dilutions required (100 vs 30 %), this demonstrates that ST8, ST22 and ST93 contain a higher proportion of highly toxic isolates than ST239 and ST36. When comparing the median difference in cytotoxicity between STs, we observe that ST22 are significantly more cytotoxic than ST8 isolates (*P*=0.025). However, no difference in cytotoxicity is observed between ST22 and ST93, ST93 and ST8 or ST239 and ST36. However, what was equally striking from this initial analysis is the scale of the variation in the toxicity of the isolates within each ST (Fig. 1).

### Toxicity cannot be inferred solely from the MRSA sequence type

**Fig. 2.**
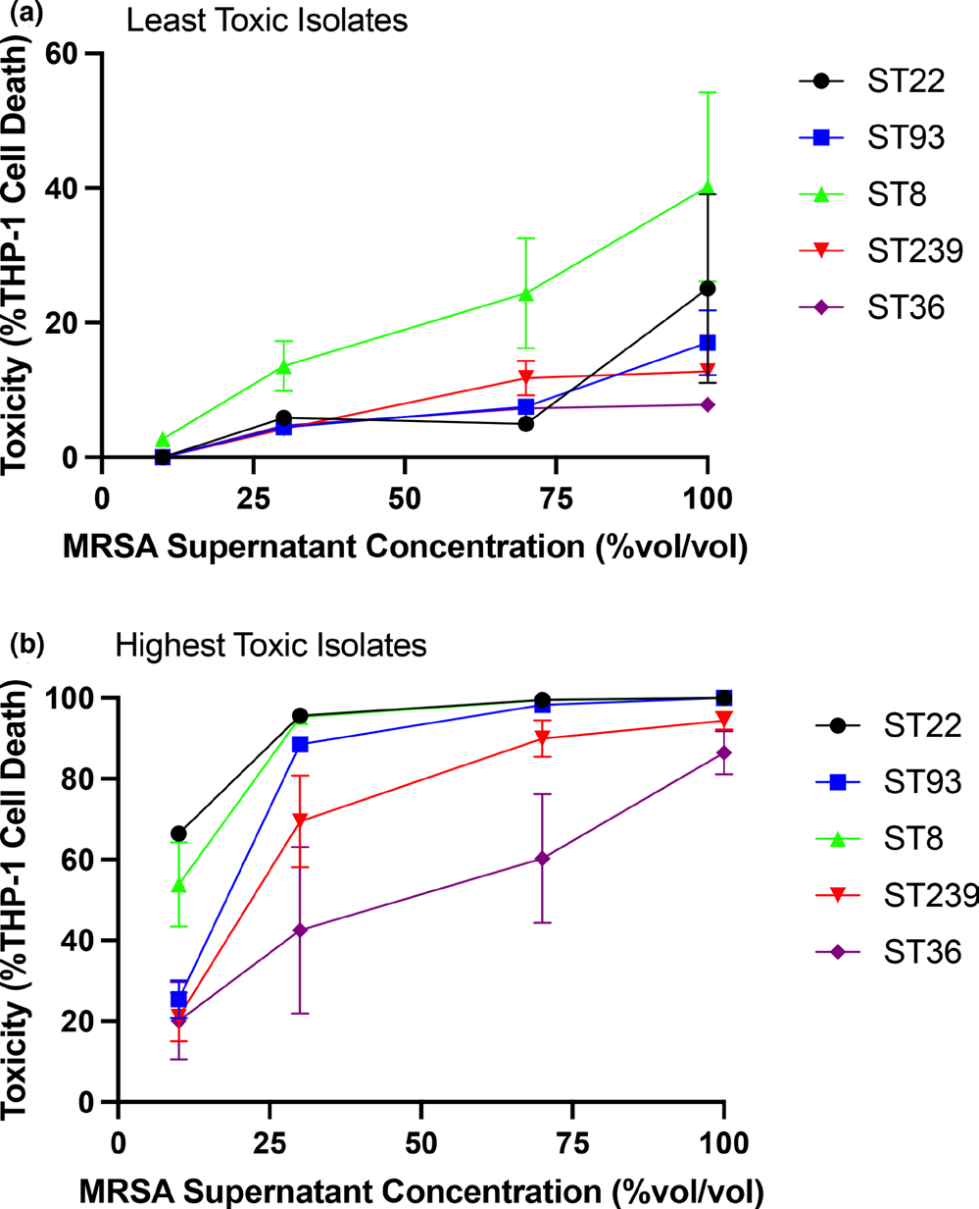
Cytotoxicity of the least and highest toxic isolates from each of the five MRSA sequence type. The supernatant of the five least (**a**) and highest (**b**) toxic isolates from each sequence type was diluted to 10, 30, or 70 % supernatant in sterile TS broth or used undiluted (100%) and the percentage cell death of THP-1 cells examined. The cytotoxicity of each isolate was quantified using three biological repeats and the data is presented as the mean and standard deviation across the five isolates.

To further compare the toxicity between STs we took the five most and five least toxic isolates from each MRSA ST and quantified their cytotoxicity over a range of dilutions of their supernatant (Fig. 2). The cytotoxicity of each supernatant dilution was measured using three biological repeats with high reproducibility (Fig. S2). The mean cytotoxicity of the five least toxic ST8 isolates was slightly higher than those from the other STs, but this was not statistically significant (*P*>0.05, Fig. 2a). This demonstrates that each of the five MRSA STs contain isolates expressing comparable low levels of cytotoxicity. Of the most toxic isolates, there were significant differences in cytotoxicity across the five STs (Figs 2 and S2). At a supernatant dilution of 10 % the ST22 and ST8 isolates were on average more toxic than the others (*P*<0.05 for each comparison, Fig. 2b). At supernatant dilution of 30%, the ST22, ST93 and ST8 were on average more toxic than the other STs (*P*<0.05 for each comparison values), while at dilution 70%, the ST36 isolates were statistically significantly less toxic than the other STs (*P*<0.05 for each comparison values).

ST22, ST93 and ST8 all contain the community-associated Type IV SCC*mec* element, which prior to this study would have led to them being referred to as hypervirulent [9, 25]. By comparison, ST239 and ST36 isolates contain the type III and II healthcare-associated SCC*mec* elements, respectively. While the ST8 and ST22 collections did contain the most toxic isolates, the most toxic ST93 isolates were no more toxic than those from the ST239 collection but was statistically more cytotoxic than the most toxic isolates of ST36 when compared at 70 % dilution (ST93 vs ST36 *P*=0.043) and at 30 % dilution (*P*=0.017). (Figs 2b and S2b). This may be a result of differential expression of cytotoxins to which our cell line is not susceptible. However, the ST93 collection did contain a higher proportion of highly toxic isolates than the ST239 and ST36 collections, which is what we observed with the other type IV SCC*mec* carrying MRSA STs studied here (Fig. 1). This observation aligns with earlier work demonstrating that the type IV element has less of a down-regulating effect on toxicity compared to the larger hospital associated SCC*mec* types [26].

### Agr mutations alone do not explain all low toxic isolates

**Fig. 3.**
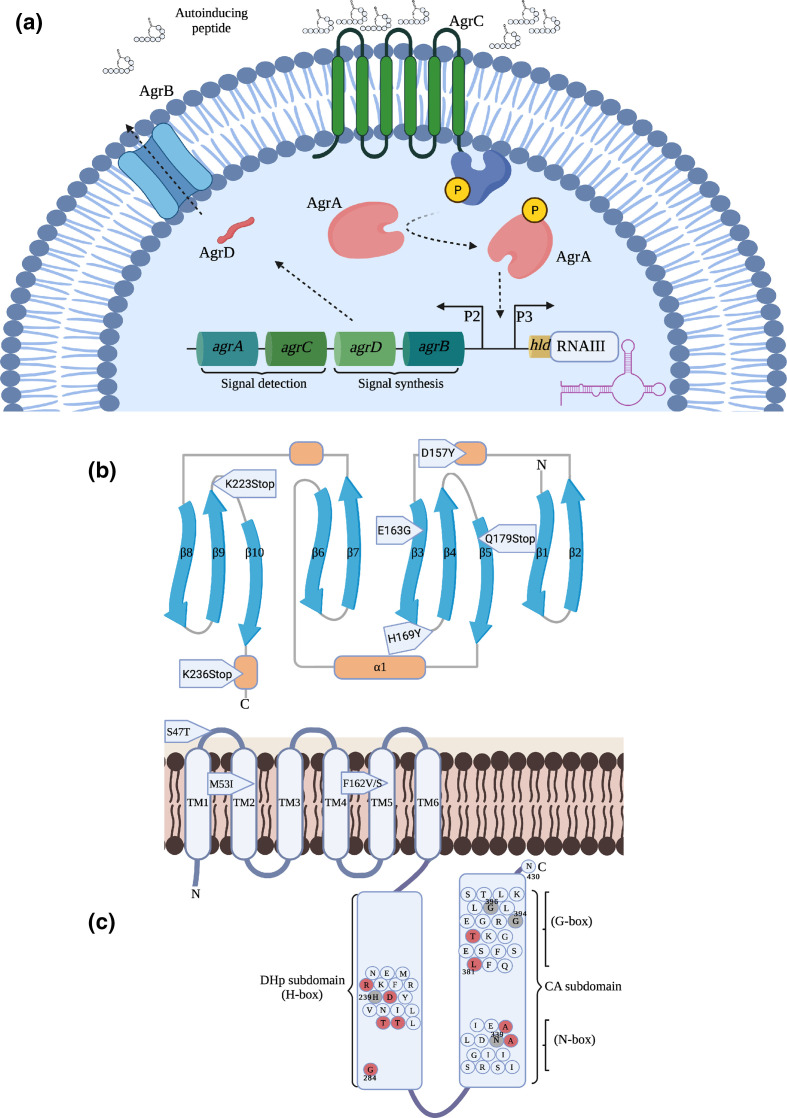
Accessory gene regulatory (Agr) system of *S. aureus* labelled with amino acid substitutions associated with reduced cytotoxicity. (**a**) The Agr locus consists of two divergent transcripts driven by the P2 and P3 promoters. P2 drives the expression of the quorum sensing systems consisting of the signal synthesis (*agrBD*) and signal detection (*agrAC*) genes. AgrB and AgrD cooperative to process and secrete autoinducing peptides (AIPs) which are sensed by the polytopic transmembrane protein, AgrC. AgrCA function as a two-component signal transduction system with AgrC phosphorylating AgrA resulting in a conformational change promoting DNA binding to the intergenic region between P2 and P3 driving their expression. The effector molecule of the Agr system, RNAIII, is expressed from P3 resulting in a shift in virulence gene expression, namely enhanced cytolytic toxin expression. (**b**) The C-terminal DNA binding domain of AgrA is shown as a ten-stranded elongated β-β-β sandwich, where the β-strands are shown in blue, helices shown in orange and loop regions shown in grey. Mutation associated with reduced toxicity are highlighted in the specific regions of the protein in which they occur. (**c**) The transmembrane sensor and cytoplasmic histidine kinase domains of AgrC are highlighted. The central histidine residue (H239) within the H-box of the DHp subdomain and the CA subdomain N-box asparagine (N339) and glycine residues of the G box (G394 and G396) are indicated. Residues labelled in red have been identified in this study to be associated with reduced cytotoxicity.

To understand the molecular mechanisms behind the observed variation in cytotoxin production, we sought to examine the impact of sequence variability within the Agr regulatory locus on this phenotype. AgrC and AgrA represent the sensor kinase and response regulator of the Agr system, respectively (Fig. 3a), and mutations within the genes encoding these key proteins are frequently associated with reduced toxin production by clinical isolates (Fig. 3a) [16]. As the genome sequences were available for all the isolates studied here, we interrogated these and found that of the 475 isolates, 14 isolates had non-synonymous mutations in *agrA* (Table 1) and 35 isolates had non-synonymous mutations in *agrC* (Table 2). The location of each of the amino acid changes inferred by these mutations is indicated in Tables 1 and 2 and have been mapped onto a representation of each protein (AgrA, Fig. 3b; AgrC, Fig. 3c) where the critically active regions are indicated.

**Table 1.** Comparison of mutations and amino acid substitutions (AA-sub) identified in the accessory gene regulator A (*agrA*) gene and cytotoxicity of MRSA isolates

ST/Strain ID	Mutation / AA-sub	Description	Cell death (%)
**ST22**			
ASARM205	Y95H	Unknown	45
ASARM93	E163G	Glutamic residue lies within beta-sheet 3, important in beta-beta-beta sandwich formation, involved in salt bridge formation	9
ASARM207	K223Stop	Dysfunctional, truncated AgrA	5
ASARM128	K236Stop	Dysfunctional, truncated AgrA	3
**ST93**			
Sa_TPS3105	Frameshift I156, T178	Dysfunctional, truncated AgrA	6
**ST8**			
MR026	(-t)2 149 463	Dysfunctional, frameshift and predicted premature stop codon at 173	12
**ST239**			
AGT9	A47D	Unknown	6
MAL119	S139I	Functional	91
**ST30**			
EOE120	D157Y	Important in formation of elongated beta-beta-beta fold and salt bridge formation	4
ASARM63	H169Y	Dysfunctional, His residue is essential for DNA binding	5
EOE176	Q179Stop codon	Dysfunctional, truncated AgrA	5
EOE161	Q179Stop codon	Dysfunctional, truncated AgrA	5
EOE171	Q179Stop codon	Dysfunctional, truncated AgrA	5
EOE169	Q179Stop codon	Dysfunctional, truncated AgrA	6

**Table 2.** Comparison of mutations and amino acid substitutions (AA-sub) identified in the accessory gene regulator C (*agrC*) gene and cytotoxicity of MRSA isolates

ST/Strain ID	Mutation / AA-sub	Description	Cell death (%)
**ST22**			
ASARM204	S47T and V367I	Mutation in extracellular loop; mutation in cytoplasmic c-terminal domain	5
ASARM84	M53I	Unknown; mutation in membrane spanning region	5
ASARM224	A57V	Functional	91
ASARM217	A57V	Functional	92
ASARM208	A57V	Functional	97
ASARM61	A57V	Functional	97
ASARM200	Y121H and Q202H	Functional	92
ASARM201	Y121H and Q202H	Functional	93
ASARM154	F162V	Unknown; mutation in membrane spanning region	13
ASARM97	A340V	Mutation of highly conserved residue in N-box CA kinase subdomain	14
**ST93**			
Sa_TPS3155	Y71H	Unknown; mutation in membrane spanning region	42
Sa_TPS3165	F162S	Unknown; mutation in membrane spanning region	15
Sa_TPS3167	M20I	Unknown; mutation in membrane spanning region	56
Sa_TPS3148	R235C	Mutation of conserved residue in H-box in DHp subdomain	6
Sa_TPS3151	D240N	Mutation of conserved residue in H-box in DHp subdomain	12
Sa_TPS3161	G284D	Mutation in histidine kinase domain -predicted to be dysfunctional	9
**ST8**			
MR030	(-t)2 146 811	Deletion (-t;2146811) within intergenic region between P2/P3 agr promoter	64
MR081	(-t)2 146 811	Deletion (-t;2146811) within intergenic region between P2/P3 agr promoter	23
MR083	(-t)2 146 811	Deletion (-t;2146811) within intergenic region between P2/P3 agr promoter	65
USFL093	Y32T	Functional	95
MR065	L381F	Mutation of non-conserved residue in G-box CA kinase subdomain	35
**ST239**			
GRE4	T246M	Mutation of conserved residue in H-box in DHp subdomain	5
ICP5062	T247I	Mutation of conserved residue in H-box in DHp subdomain	6
HU5	I311T; A343T	Dysfunctional; I311T/A343T resulted in delayed RNAIII activity	15
HU11	I311T; A343T	Dysfunctional; I311T/A343T resulted in delayed RNAIII activity	18
DEU29	I311T; A343T	Dysfunctional; I311T/A343T resulted in delayed RNAIII activity	19
DEU37	I311T; A343T	Dysfunctional; I311T/A343T resulted in delayed RNAIII activity	19
HU6	I311T; A343T	Dysfunctional; I311T/A343T resulted in delayed RNAIII activity	120
DEU20	I311T; A343T	Dysfunctional; I311T/A343T resulted in delayed RNAIII activity	21
DEU9	I311T; A343T	Dysfunctional; I311T/A343T resulted in delayed RNAIII activity	20
DEU17	I311T; A343T	Dysfunctional; I311T/A343T resulted in delayed RNAIII activity	16
HU16	I311T; A343T	Dysfunctional; I311T/A343T resulted in delayed RNAIII activity	18
CHI61	M326T	Functional	80
**ST36**			
EOE173	T247I	Mutation of conserved residue in H-box in DHp subdomain	6
EOE096	T388I	Mutation of conserved residue in G-box CA subdomain	7

Given the major role the Agr system plays in regulating cytotoxin production in *S. aureus* we examined what proportion of low toxic isolates could be explained by these Agr mutations. We set an arbitrary threshold where we considered any isolate that killed less than 20 % of the cells as ‘low toxicity’, and any that killed more than 80 % as ‘high toxicity’. Of the 475 isolates, 17.7 % (*n*=84) of the isolates were categorised as low toxicity and of these 34 (40.5 %) had non-synonymous mutations in the *agr* locus. While of the high toxicity isolates (*n*=215 (45.2 %)) eight had non-synonymous mutations in the *agr* locus, suggesting that these amino acids are not critical to the activity of the Agr system (Tables 1 and 2). Of the 34 low toxicity isolates with mutations in the Agr locus, while we cannot assume these mutations are causative without making a series of isogenic mutants, having mapped them to the functional regions of the proteins (Fig. 3b, c) we can hypothesise those mutations that are likely to be causative of the low toxicity phenotype.

#### Assessment of *agrA* mutations

The AgrA protein is part of the LytR family of DNA-binding response regulators that modulate virulence determinants in several pathogenic bacteria [27]. This transcription factor contains the LytTR domain which binds with high affinity to both P2 and P3 promoter regions of the *agr* locus upregulating toxin production (Fig. 3a) [16, 28]. The structure of the C-terminal LytTR DNA binding domain of AgrA (residues 137–238) in complex with a DNA duplex has been solved [29], highlighting the formation of an intricate ten-stranded elongated β-β-β sandwich (Fig. 3b). Key residues within three loop regions extending from the β-sheets makes contact with the major and intervening minor groove of its DNA target resulting in increased transcription from the P2 and P3 promoters and activation of the Agr system [29]. The mutations in eight of the low toxicity isolates resulted in premature truncation of the AgrA protein, which caused a loss of a functional region, and as such can be considered causative of the low toxicity phenotype (Table 1, Fig. 3b). Two of the other low toxicity isolates (ASARM93 and EOE120) had substitution mutations (E163G and D157Y, respectively) within the critical β-β-β sandwich. Mutation E163G occurs within β-strand three which forms the centre of the LytTR domain and plays a role in salt-bridge formation with H174 [29]. Mutation D157Y is located within an α-helix between strands β2 and β3 and forms a salt bridge interaction with both H208 located on helix between β5 and β6 and E141 positioned on the beginning of strand β1. These salt bridge interactions stabilise the LytTR β-β-β fold, and our data indicates that mutations within these residues impairs Agr activity and results in reduced toxicity.

Lastly, isolate ASARM63 had a mutation conferring a H169Y change, which results in the loss of a histidine residue critical to the DNA binding activity of AgrA [32]. Only one low toxic *agrA* mutant contained a substitution in a region with no ascribed function, and that was AGT9 which had an A47D change (not shown in Fig. 3b).

#### Assessment of *agrC* mutations

The AgrC protein is composed of a highly variable polytopic transmembrane sensor which relays auto-inducing peptide (AIP) mediated signals to the highly conserved cytoplasmic histidine kinase (HK) domain (Fig. 3a, c) [30]. The AgrC HK domain is composed of two subdomains; the catalytic ATP-binding (CA) domain which promotes the autophosphorylation of the central histidine (His239) residue within the H-box of the dimerized histidine phosphotransfer (DHp) domain (Fig. 3c) [30–32]. The CA subdomain N-box asparagine (N339) and glycine residues situated within the G-box (G394 and G396) are considered essential for ATP binding and Agr activity [33, 34]. Importantly, alanine 340 and threonine 388 are conserved residues within this subdomain [32]. Two low toxic isolates (ASARM97 (A340V) and EOE096 (T388I)) contain substitutions in the CA kinase subdomain region of AgrC, and as such may be causative of the low toxic phenotype. Six low toxic isolates (Sa_TPS3148, Sa_TPS3151, Sa_TPS3161, GRE4, ICP5062 and EOE173) occur in conserved residues within the H-box of the DHp subdomain and as such, are likely to be causative of the low toxicity phenotype. Nine isolates, all ST239 belonging to the Turkish lineage [35], contained a double substitution of I311T and A343T, and in previous work we have functionally verified that these mutations result in a significant delay to RNAIII activation [18]. Molecular modelling has previously indicated that these mutations prevent AgrC dimerization and access to the ATP-binding pocket required for Agr activity which explains the low toxic phenotype [36].

Of the low toxic isolates with *agrC* mutations, four (ASARM204, ASARM84, ASARM154 and Sa_TPS3165) contain substitutions within either the extracellular or cytoplasmic regions of the protein with no ascribed function, and so we cannot claim with a level of confidence that these are causative of the low toxic phenotype.

This study adds to the growing literature indicating that cytotoxin regulation is highly complex in *S. aureus* and confirms that there are still undiscovered mechanisms at play that modulate this major virulence phenotype. This works reinforces the importance of Agr mutations in *S. aureus* toxicity. The data generated here provides a clearer understanding of the relationship between Agr mutations and toxicity, which may be exploited for future anti-virulence drug design. However, considering that close to 60 % of low toxicity isolates had no Agr mutations demonstrates that numerous regulatory mechanisms await discovery, and work dedicated to unravelling the regulatory circuits controlling the toxicity of *S. aureus* is ongoing. This also suggests that use of the term ‘Agr dysfunction’ should be used with consideration of the fact that for many of low toxicity clinical isolates the Agr system is likely to be functional.

What this work primarily highlights is the care and consideration needed when inferring terms like ‘hypervirulence’ to an isolate based on its sequence type. There can be no doubt that CA-MRSA lineages have recently emerged and are highly successful. Whether this is a result of ‘hypervirulence’ as opposed to an enhanced ability to transmit amongst otherwise healthy individuals needs further investigation. From the perspective of cytolytic toxin production by MRSA, the level of variability is significant and likely to play a major role in the outcome of *S. aureus* disease.

## Supplementary Data

Supplementary material 1
