# Supplementary material for: Significant variability exists in the cytotoxicity of global methicillin-resistant Staphylococcus aureus lineages
Source: Microbiology (Reading). 2021 Dec 20;167(12):001119. doi: 10.1099/mic.0.001119 (PMC8744995; doi:10.1099/mic.0.001119)
Supplement: Supplementary material 1 [file mic-167-1119-s001.pdf]

Supplementary Material

Significant Variability exists in the Cytotoxicity of Global Methicillin-resistant *Staphylococcus aureus* Lineages.

Maisem Laabei[1]*, Sharon J. Peacock[2], Beth Blane[2], Sarah L. Baines[3], Benjamin P. Howden[3,4], Timothy P. Stinear[3] and Ruth C. Massey[5]*.

1: Department of Biology and Biochemistry, University of Bath, Bath, BA2 7AY, UK
2: Department of Medicine, Addenbrooke's Hospital, University of Cambridge, Hills Road, Box 157, Cambridge, CB2 0QQ, UK
3: Department of Microbiology and Immunology, University of Melbourne at the Peter Doherty Institute for Infection and Immunity, Melbourne, Australia
4: Microbiological Diagnostic Unit Public Health Laboratory, University of Melbourne at the Peter Doherty Institute for Infection and Immunity, Melbourne, Australia
5: School of Cellular and Molecular Medicine, University of Bristol, Bristol, BS8 1TD, UK.

* For correspondence, please email ml418@bath.ac.uk; ruth.massey@bristol.ac.uk

Supplementary Table 1: List of clinical isolates used in this study

Clinical Isolate	Sequence Type	Country of Origin	Disease Status	Year
ASARM100	ST22	UK	Bacteraemia	2007
ASARM101	ST22	UK	Bacteraemia	2007
ASARM102	ST22	UK	Bacteraemia	2007
ASARM103	ST22	UK	Bacteraemia	2007
ASARM105	ST22	UK	Bacteraemia	2007
ASARM107	ST22	UK	Bacteraemia	2007
ASARM108	ST22	UK	Bacteraemia	2007
ASARM109	ST22	UK	Bacteraemia	2007
ASARM110	ST22	UK	Bacteraemia	2007
ASARM114	ST22	UK	Bacteraemia	2007
ASARM116	ST22	UK	Bacteraemia	2007
ASARM117	ST22	UK	Bacteraemia	2007
ASARM118	ST22	UK	Bacteraemia	2007
ASARM119	ST22	UK	Bacteraemia	2007
ASARM120	ST22	UK	Bacteraemia	2007
ASARM121	ST22	UK	Bacteraemia	2007
ASARM122	ST22	UK	Bacteraemia	2007
ASARM124	ST22	UK	Bacteraemia	2007
ASARM125	ST22	UK	Bacteraemia	2007
ASARM126	ST22	UK	Bacteraemia	2007
ASARM127	ST22	UK	Bacteraemia	2007
ASARM128	ST22	UK	Bacteraemia	2007
ASARM132	ST22	UK	Bacteraemia	2007
ASARM133	ST22	UK	Bacteraemia	2007
ASARM134	ST22	UK	Bacteraemia	2007
ASARM135	ST22	UK	Bacteraemia	2007
ASARM136	ST22	UK	Bacteraemia	2007
ASARM137	ST22	UK	Bacteraemia	2007
ASARM138	ST22	UK	Bacteraemia	2007
ASARM139	ST22	UK	Bacteraemia	2007
ASARM140	ST22	UK	Bacteraemia	2007
ASARM141	ST22	UK	Bacteraemia	2007
ASARM142	ST22	UK	Bacteraemia	2007
ASARM143	ST22	UK	Bacteraemia	2007
ASARM144	ST22	UK	Bacteraemia	2007
ASARM145	ST22	UK	Bacteraemia	2007
ASARM148	ST22	UK	Bacteraemia	2008
ASARM153	ST22	UK	Bacteraemia	2008
ASARM154	ST22	UK	Bacteraemia	2008
ASARM155	ST22	UK	Bacteraemia	2008

ASARM160	ST22	UK	Bacteraemia	2008
ASARM162	ST22	UK	Bacteraemia	2008
ASARM163	ST22	UK	Bacteraemia	2008
ASARM164	ST22	UK	Bacteraemia	2008
ASARM165	ST22	UK	Bacteraemia	2008
ASARM166	ST22	UK	Bacteraemia	2008
ASARM167	ST22	UK	Bacteraemia	2008
ASARM168	ST22	UK	Bacteraemia	2008
ASARM169	ST22	UK	Bacteraemia	2008
ASARM170	ST22	UK	Bacteraemia	2008
ASARM171	ST22	UK	Bacteraemia	2008
ASARM172	ST22	UK	Bacteraemia	2008
ASARM176	ST22	UK	Bacteraemia	2009
ASARM177	ST22	UK	Bacteraemia	2009
ASARM179	ST22	UK	Bacteraemia	2009
ASARM181	ST22	UK	Bacteraemia	2009
ASARM183	ST22	UK	Bacteraemia	2009
ASARM184	ST22	UK	Bacteraemia	2009
ASARM191	ST22	UK	Bacteraemia	2009
ASARM193	ST22	UK	Bacteraemia	2009
ASARM195	ST22	UK	Bacteraemia	2009
ASARM196	ST22	UK	Bacteraemia	2009
ASARM199	ST22	UK	Bacteraemia	2009
ASARM200	ST22	UK	Bacteraemia	2010
ASARM201	ST22	UK	Bacteraemia	2010
ASARM203	ST22	UK	Bacteraemia	2010
ASARM204	ST22	UK	Bacteraemia	2010
ASARM205	ST22	UK	Bacteraemia	2010
ASARM207	ST22	UK	Bacteraemia	2010
ASARM208	ST22	UK	Bacteraemia	2010
ASARM209	ST22	UK	Bacteraemia	2010
ASARM211	ST22	UK	Bacteraemia	2010
ASARM212	ST22	UK	Bacteraemia	2010
ASARM217	ST22	UK	Bacteraemia	2011
ASARM220	ST22	UK	Bacteraemia	2011
ASARM221	ST22	UK	Bacteraemia	2011
ASARM222	ST22	UK	Bacteraemia	2012
ASARM223	ST22	UK	Bacteraemia	2012
ASARM224	ST22	UK	Bacteraemia	2012
ASARM59	ST22	UK	Bacteraemia	2006
ASARM61	ST22	UK	Bacteraemia	2006
ASARM62	ST22	UK	Bacteraemia	2006
ASARM64	ST22	UK	Bacteraemia	2006

ASARM65	ST22	UK	Bacteraemia	2006
ASARM67	ST22	UK	Bacteraemia	2006
ASARM68	ST22	UK	Bacteraemia	2006
ASARM69	ST22	UK	Bacteraemia	2006
ASARM70	ST22	UK	Bacteraemia	2006
ASARM71	ST22	UK	Bacteraemia	2006
ASARM72	ST22	UK	Bacteraemia	2006
ASARM73	ST22	UK	Bacteraemia	2006
ASARM74	ST22	UK	Bacteraemia	2006
ASARM75	ST22	UK	Bacteraemia	2006
ASARM76	ST22	UK	Bacteraemia	2006
ASARM77	ST22	UK	Bacteraemia	2006
ASARM79	ST22	UK	Bacteraemia	2006
ASARM80	ST22	UK	Bacteraemia	2006
ASARM83	ST22	UK	Bacteraemia	2006
ASARM84	ST22	UK	Bacteraemia	2006
ASARM86	ST22	UK	Bacteraemia	2006
ASARM87	ST22	UK	Bacteraemia	2006
ASARM89	ST22	UK	Bacteraemia	2006
ASARM93	ST22	UK	Bacteraemia	2006
ASARM95	ST22	UK	Bacteraemia	2006
ASARM96	ST22	UK	Bacteraemia	2006
ASARM97	ST22	UK	Bacteraemia	2006
ASARM99	ST22	UK	Bacteraemia	2007
ASARMLT1	ST22	UK	Bacteraemia	2008
ASARMLT2	ST22	UK	Bacteraemia	2008
ASARMLT3	ST22	UK	Bacteraemia	2008
Sa_TPS3105	ST93	Australia	Bacteraemia	2005
Sa_TPS3148	ST93	Australia	Bacteraemia	2007
Sa_TPS3161	ST93	Australia	SSTI	2008
Sa_TPS3151	ST93	Australia	SSTI	2008
Sa_TPS3165	ST93	Australia	Carriage	2008
Sa_TPS3150	ST93	Australia	Sputum	2007
Sa_TPS3171	ST93	Australia	Carriage	2008
Sa_TPS3155	ST93	Australia	Carriage	2008
Sa_TPS3118	ST93	Australia	not recorded	not recorded
Sa_TPS3167	ST93	Australia	endotrachael tube	2008
Sa_TPS3162	ST93	Australia	Carriage	2003
Sa_TPS3169	ST93	Australia	SSTI	2008
Sa_TPS3106	ST93	Australia	Carriage	2008
Sa_TPS3145	ST93	Australia	SSTI	2006
Sa_TPS3158	ST93	Australia	SSTI	2008
Sa_TPS3183	ST93	Australia	not recorded	2007
Sa_TPS3134	ST93	Australia	SSTI	2000

Sa_TPS3137	ST93	Australia	SSTI	2000
Sa_TPS3181	ST93	Australia	SSTI	2008
Sa_TPS3026	ST93	Australia	Bacteraemia	2004
Sa_TPS3142	ST93	Australia	Bacteraemia	2005
Sa_TPS3164	ST93	Australia	Carriage	2008
Sa_TPS3153	ST93	Australia	Carriage	2008
Sa_TPS3104	ST93	Australia	Carriage	2009
Sa_TPS3139	ST93	Australia	SSTI	2000
Sa_TPS3149	ST93	Australia	Carriage	2007
Sa_TPS3138	ST93	Australia	SSTI	2000
Sa_TPS3160	ST93	Australia	SSTI	2008
Sa_TPS3185	ST93	Australia	Carriage	2003
Sa_TPS3176	ST93	Australia	SSTI	2009
Sa_TPS3144	ST93	Australia	SSTI	2006
Sa_TPS3174	ST93	Australia	SSTI	2009
Sa_TPS3146	ST93	Australia	SSTI	2006
Sa_TPS3133	ST93	Australia	Carriage	2000
Sa_TPS3132	ST93	Australia	not recorded	2000
Sa_TPS3152	ST93	Australia	pneumoniae	2008
Sa_TPS3135	ST93	Australia	Carriage	2000
Sa_TPS3173	ST93	Australia	Carriage	2009
Sa_TPS3140	ST93	Australia	SSTI	2000
Sa_TPS3136	ST93	Australia	Bacteraemia	2000
Sa_TPS3188	ST93	Australia	not recorded	1992
Sa_TPS3166	ST93	Australia	Carriage	2008
Sa_TPS3178	ST93	Australia	Pleural fluid	2008
Sa_TPS3157	ST93	Australia	SSTI	2008
Sa_TPS3168	ST93	Australia	SSTI	2008
Sa_TPS3189	ST93	Australia	not recorded	1992
Sa_TPS3154	ST93	Australia	SSTI	2008
Sa_TPS3163	ST93	Australia	SSTI	2005
Sa_TPS3184	ST93	Australia	Carriage	1995
Sa_TPS3182	ST93	Australia	not recorded	2008
Sa_TPS3147	ST93	Australia	SSTI	2006
Sa_TPS3177	ST93	Australia	Bacteraemia	2008
Sa_TPS3159	ST93	Australia	SSTI	2008
Sa_TPS3156	ST93	Australia	Carriage	2008
Sa_TPS3180	ST93	Australia	not recorded	not recorded
Sa_TPS3186	ST93	Australia	Carriage	2003
Sa_TPS3179	ST93	Australia	Carriage	2008
Sa_TPS3187	ST93	Australia	Carriage	1996
MR005	ST8	USA	Bacteraemia	2009-2011
MR007	ST8	USA	Bacteraemia	2009-2011
MR018	ST8	USA	Bacteraemia	2009-2011
MR019	ST8	USA	Bacteraemia	2009-2011
MR021	ST8	USA	Bacteraemia	2009-2011

MR022	ST8	USA	Bacteraemia	2009-2011
MR023	ST8	USA	Bacteraemia	2009-2011
MR025	ST8	USA	Bacteraemia	2009-2011
MR026	ST8	USA	Bacteraemia	2009-2011
MR027	ST8	USA	Bacteraemia	2009-2011
MR029	ST8	USA	Bacteraemia	2009-2011
MR030	ST8	USA	Bacteraemia	2009-2011
MR031	ST8	USA	Bacteraemia	2009-2011
MR035	ST8	USA	Bacteraemia	2009-2011
MR036	ST8	USA	Bacteraemia	2009-2011
MR039	ST8	USA	Bacteraemia	2009-2011
MR047	ST8	USA	Bacteraemia	2009-2011
MR051	ST8	USA	Bacteraemia	2009-2011
MR060	ST8	USA	Bacteraemia	2009-2011
MR063	ST8	USA	Bacteraemia	2009-2011
MR064	ST8	USA	Bacteraemia	2009-2011
MR065	ST8	USA	Bacteraemia	2009-2011
MR072	ST8	USA	Bacteraemia	2009-2011
MR073	ST8	USA	Bacteraemia	2009-2011
MR074	ST8	USA	Bacteraemia	2009-2011
MR077	ST8	USA	Bacteraemia	2009-2011
MR078	ST8	USA	Bacteraemia	2009-2011
MR081	ST8	USA	Bacteraemia	2009-2011
MR083	ST8	USA	Bacteraemia	2009-2011
MR084	ST8	USA	Bacteraemia	2009-2011
MR087	ST8	USA	Bacteraemia	2009-2011
MR090	ST8	USA	Bacteraemia	2009-2011
MR091	ST8	USA	Bacteraemia	2009-2011
MR096	ST8	USA	Bacteraemia	2009-2011
MR107	ST8	USA	Bacteraemia	2009-2011
MR110	ST8	USA	Bacteraemia	2009-2011
USFL008	ST8	USA	Carriage	2009-2011
USFL009	ST8	USA	Carriage	2009-2011
USFL012	ST8	USA	Carriage	2009-2011
USFL028	ST8	USA	Carriage	2009-2011
USFL042	ST8	USA	Carriage	2009-2011
USFL061	ST8	USA	Carriage	2009-2011
USFL063	ST8	USA	Carriage	2009-2011
USFL074	ST8	USA	Carriage	2009-2011
USFL077	ST8	USA	Carriage	2009-2011
USFL082	ST8	USA	Carriage	2009-2011
USFL093	ST8	USA	Carriage	2009-2011
USFL119	ST8	USA	Carriage	2009-2011
USFL130	ST8	USA	Carriage	2009-2011
USFL141	ST8	USA	Carriage	2009-2011
USFL153	ST8	USA	Carriage	2009-2011

USFL156	ST8	USA	Carriage	2009-2011
USFL166	ST8	USA	Carriage	2009-2011
USFL167	ST8	USA	Carriage	2009-2011
USFL169	ST8	USA	Carriage	2009-2011
USFL182	ST8	USA	Carriage	2009-2011
USFL200	ST8	USA	Carriage	2009-2011
USFL211	ST8	USA	Carriage	2009-2011
USFL213	ST8	USA	Carriage	2009-2011
USFL224	ST8	USA	Carriage	2009-2011
USFL225	ST8	USA	Carriage	2009-2011
USFL230	ST8	USA	Carriage	2009-2011
USFL231	ST8	USA	Carriage	2009-2011
USFL243	ST8	USA	Carriage	2009-2011
USFL248	ST8	USA	Carriage	2009-2011
USFL259	ST8	USA	Carriage	2009-2011
USFL263	ST8	USA	Carriage	2009-2011
USFL267	ST8	USA	Carriage	2009-2011
USFL269	ST8	USA	Carriage	2009-2011
USFL271	ST8	USA	Carriage	2009-2011
USFL272	ST8	USA	Carriage	2009-2011
USFL302	ST8	USA	Carriage	2009-2011
USFL303	ST8	USA	Carriage	2009-2011
USFL304	ST8	USA	Carriage	2009-2011
USFL016	ST8	USA	SSTI	2009-2011
USFL018	ST8	USA	SSTI	2009-2011
USFL020	ST8	USA	SSTI	2009-2011
USFL021	ST8	USA	SSTI	2009-2011
USFL034	ST8	USA	SSTI	2009-2011
USFL035	ST8	USA	SSTI	2009-2011
USFL036	ST8	USA	SSTI	2009-2011
USFL039	ST8	USA	SSTI	2009-2011
USFL056	ST8	USA	SSTI	2009-2011
USFL057	ST8	USA	SSTI	2009-2011
USFL059	ST8	USA	SSTI	2009-2011
USFL069	ST8	USA	SSTI	2009-2011
USFL095	ST8	USA	SSTI	2009-2011
USFL097	ST8	USA	SSTI	2009-2011
USFL103	ST8	USA	SSTI	2009-2011
USFL110	ST8	USA	SSTI	2009-2011
USFL111	ST8	USA	SSTI	2009-2011
USFL113	ST8	USA	SSTI	2009-2011
USFL136	ST8	USA	SSTI	2009-2011
USFL137	ST8	USA	SSTI	2009-2011
USFL138	ST8	USA	SSTI	2009-2011
USFL139	ST8	USA	SSTI	2009-2011
USFL149	ST8	USA	SSTI	2009-2011

USFL152	ST8	USA	SSTI	2009-2011
USFL158	ST8	USA	SSTI	2009-2011
USFL159	ST8	USA	SSTI	2009-2011
USFL160	ST8	USA	SSTI	2009-2011
USFL162	ST8	USA	SSTI	2009-2011
USFL164	ST8	USA	SSTI	2009-2011
USFL165	ST8	USA	SSTI	2009-2011
USFL173	ST8	USA	SSTI	2009-2011
USFL174	ST8	USA	SSTI	2009-2011
USFL194	ST8	USA	SSTI	2009-2011
USFL198	ST8	USA	SSTI	2009-2011
USFL218	ST8	USA	SSTI	2009-2011
USFL219	ST8	USA	SSTI	2009-2011
USFL220	ST8	USA	SSTI	2009-2011
USFL221	ST8	USA	SSTI	2009-2011
USFL222	ST8	USA	SSTI	2009-2011
USFL223	ST8	USA	SSTI	2009-2011
USFL237	ST8	USA	SSTI	2009-2011
USFL239	ST8	USA	SSTI	2009-2011
USFL240	ST8	USA	SSTI	2009-2011
USFL250	ST8	USA	SSTI	2009-2011
USFL255	ST8	USA	SSTI	2009-2011
USFL256	ST8	USA	SSTI	2009-2011
USFL258	ST8	USA	SSTI	2009-2011
USFL273	ST8	USA	SSTI	2009-2011
USFL274	ST8	USA	SSTI	2009-2011
USFL276	ST8	USA	SSTI	2009-2011
USFL277	ST8	USA	SSTI	2009-2011
USFL279	ST8	USA	SSTI	2009-2011
USFL282	ST8	USA	SSTI	2009-2011
USFL319	ST8	USA	SSTI	2009-2011
USFL320	ST8	USA	SSTI	2009-2011
USFL326	ST8	USA	SSTI	2009-2011
USFL327	ST8	USA	SSTI	2009-2011
USFL330	ST8	USA	SSTI	2009-2011
USFL339	ST8	USA	SSTI	2009-2011
USFL341	ST8	USA	SSTI	2009-2011
ASARM112	ST36	UK	Bacteraemia	2003
ASARM156	ST36	UK	Bacteraemia	2004
ASARM161	ST36	UK	Bacteraemia	2004
ASARM180	ST36	UK	Bacteraemia	2005
ASARM185	ST36	UK	Bacteraemia	2005
ASARM190	ST36	UK	Bacteraemia	2005
ASARM197	ST36	UK	Bacteraemia	2005
ASARM210	ST36	UK	Bacteraemia	2006
ASARM213	ST36	UK	Bacteraemia	2006

ASARM63	ST36	UK	Bacteraemia	2002
ASARM88	ST36	UK	Bacteraemia	2002
ASARM90	ST36	UK	Bacteraemia	2002
ASARM92	ST36	UK	Bacteraemia	2002
EOE 3	ST36	UK	Bacteraemia	2007
EOE 23	ST36	UK	Bacteraemia	2008
EOE 30	ST36	UK	Bacteraemia	1995
EOE 35	ST36	UK	Bacteraemia	1999
EOE 41	ST36	UK	Bacteraemia	2001
EOE 42	ST36	UK	Bacteraemia	2001
EOE 45	ST36	UK	Bacteraemia	2001
EOE 52	ST36	UK	Bacteraemia	1995
EOE 54	ST36	UK	Bacteraemia	1998
EOE 57	ST36	UK	Bacteraemia	1996
EOE 61	ST36	UK	Bacteraemia	1994
EOE 65	ST36	UK	Bacteraemia	1994
EOE 72	ST36	UK	Bacteraemia	1994
EOE 73	ST36	UK	Bacteraemia	1994
EOE 78	ST36	UK	Bacteraemia	1995
EOE 83	ST36	UK	Bacteraemia	1995
EOE 84	ST36	UK	Bacteraemia	1995
EOE 86	ST36	UK	Bacteraemia	1995
EOE 88	ST36	UK	Bacteraemia	1995
EOE 89	ST36	UK	Bacteraemia	1995
EOE 90	ST36	UK	Bacteraemia	1995
EOE 91	ST36	UK	Bacteraemia	1995
EOE 94	ST36	UK	Bacteraemia	1995
EOE 96	ST36	UK	Bacteraemia	1995
EOE 97	ST36	UK	Bacteraemia	1996
EOE 98	ST36	UK	Bacteraemia	1996
EOE 99	ST36	UK	Bacteraemia	1996
EOE 100	ST36	UK	Bacteraemia	1996
EOE 101	ST36	UK	Bacteraemia	1996
EOE 102	ST36	UK	Bacteraemia	1996
EOE 103	ST36	UK	Bacteraemia	1996
EOE 104	ST36	UK	Bacteraemia	1996
EOE 105	ST36	UK	Bacteraemia	1996
EOE 106	ST36	UK	Bacteraemia	1996
EOE 122	ST36	UK	Bacteraemia	1996
EOE 125	ST36	UK	Bacteraemia	1996
EOE 129	ST36	UK	Bacteraemia	1996
EOE 130	ST36	UK	Bacteraemia	1996
EOE 137	ST36	UK	Bacteraemia	1996
EOE 140	ST36	UK	Bacteraemia	1997
EOE 154	ST36	UK	Bacteraemia	1998
EOE 155	ST36	UK	Bacteraemia	1998

EOE 158	ST36	UK	Bacteraemia	1998
EOE 118	ST36	UK	Bacteraemia	1996
EOE 198	ST36	UK	Bacteraemia	1999
EOE 205	ST36	UK	Bacteraemia	1999
EOE 220	ST36	UK	Bacteraemia	2000
EOE 120	ST36	UK	Bacteraemia	1996
EOE 225	ST36	UK	Bacteraemia	2000
EOE 233	ST36	UK	Bacteraemia	2001
EOE 234	ST36	UK	Bacteraemia	2001
EOE 237	ST36	UK	Bacteraemia	2001
EOE 268	ST36	UK	Bacteraemia	2003
EOE 269	ST36	UK	Bacteraemia	2003
EOE 274	ST36	UK	Bacteraemia	2003
EOE 275	ST36	UK	Bacteraemia	2003
EOE 276	ST36	UK	Bacteraemia	2003
EOE 29	ST36	UK	Bacteraemia	1995
EOE 161	ST36	UK	Bacteraemia	1998
EOE 162	ST36	UK	Bacteraemia	1998
EOE 163	ST36	UK	Bacteraemia	1998
EOE 165	ST36	UK	Bacteraemia	1998
EOE 166	ST36	UK	Bacteraemia	1998
EOE 167	ST36	UK	Bacteraemia	1998
EOE 169	ST36	UK	Bacteraemia	1998
EOE 171	ST36	UK	Bacteraemia	1998
EOE 173	ST36	UK	Bacteraemia	1998
EOE 174	ST36	UK	Bacteraemia	1998
EOE 175	ST36	UK	Bacteraemia	1998
EOE 176	ST36	UK	Bacteraemia	1998
EOE 208	ST36	UK	Bacteraemia	1998
EOE 126	ST36	UK	Bacteraemia	2006
EOE 229	ST36	UK	Bacteraemia	2001
2A8	ST239	Czech Republic	not recorded	2001
2HK	ST239	Czech Republic	not recorded	2001
3HK	ST239	Czech Republic	not recorded	2000
AGT1	ST239	Argentina	not recorded	1997
AGT120	ST239	Argentina	not recorded	1998
AGT67	ST239	Argentina	not recorded	1997
AGT9	ST239	Argentina	not recorded	1997
ANS46	ST239	Australia	not recorded	1982
BK2421	ST239	USA	not recorded	1996
BRA2	ST239	Brazil	not recorded	1997
BRA36	ST239	Brazil	not recorded	1997
CHI59	ST239	China	not recorded	1998
CHI61	ST239	China	not recorded	1998
CHL1	ST239	Chile	not recorded	1997
CHL151	ST239	Chile	not recorded	1998

D71	ST239	Germany	Wound	1996
DEN907	ST239	Denmark	not recorded	2001
DEU17	ST239	Turkey	Blood	2008
DEU20	ST239	Turkey	Blood	2008
DEU29	ST239	Turkey	Blood	2007
DEU37	ST239	Turkey	Blood	2007
DEU9	ST239	Turkey	Aspiration	2009
ES26	ST239	Spain	Skin	1996
FRICAR	ST239	France	not recorded	not recorded
GRE108	ST239	Greece	not recorded	1998
GRE4	ST239	Greece	not recorded	1998
H202	ST239	Thailand	Wound	2006
H211	ST239	Denmark	Lung	2006
H216	ST239	Denmark	Blood	2006
H482	ST239	Romania	Nose (carriage)	1996
HDG2	ST239	Portugal	not recorded	1992
HGSA142	ST239	Portugal	not recorded	2003
HGSA6	ST239	Portugal	not recorded	1997
HGSA9	ST239	Portugal	not recorded	1997
HSA10	ST239	Portugal	not recorded	1992
HSI216	ST239	Portugal	not recorded	1997
HU106	ST239	Hungary	not recorded	1996
HU109	ST239	Hungary	not recorded	1996
HU11	ST239	Turkey	Blood	2007
HU13	ST239	Turkey	Brain abscess	2006
HU16	ST239	Turkey	Spinal Fluid	2007
HU25	ST239	Brazil	not recorded	1993
HU5	ST239	Turkey	Catheter	2006
HU6	ST239	Turkey	Sputum	2006
HU7	ST239	Turkey	Abscess	2007
HU8	ST239	Turkey	Abscess	2006
ICP5011	ST239	Portugal	not recorded	1993
ICP5014	ST239	Portugal	not recorded	1993
ICP5062	ST239	Portugal	not recorded	1993
IU10	ST239	Turkey	Blood	2007
IU12	ST239	Turkey	Abscess	2007
IU17	ST239	Turkey	not recorded	2007
IU20	ST239	Turkey	not recorded	2007
IU4	ST239	Turkey	Sputum	2006
IU9	ST239	Turkey	Nasal swab	2007
LIT2	ST239	Lithuania	not recorded	not recorded
LIT68	ST239	Lithuania	Wound	1996
LIT76	ST239	Lithuania	Wound	1996
LIT89	ST239	Lithuania	Blood	1996
M116	ST239	Vitnam	not recorded	2004
M1229	ST239	Denmark	Lung	2009

M278	ST239	Portugal	Nose (carriage)	2005
M418	ST239	India	Nose (carriage)	2006
M705	ST239	Thailand	Lung	2007
M74	ST239	Extensive travel	not recorded	not recorded
M996	ST239	China	Lung	2008
MAL1	ST239	Malaysia	Wound	1996
MAL11	ST239	Malaysia	Wound	1996
MAL119	ST239	Malaysia	not recorded	1996
MAL215	ST239	Malaysia	not recorded	not recorded
MAL3	ST239	Malaysia	not recorded	not recorded
MAL35	ST239	Malaysia	not recorded	not recorded
MU11	ST239	Turkey	Blood	2006
Na21	ST239	Sri Lanka	not recorded	1996
P32	ST239	Poland	Lung	1996
R3J	ST239	Poland	not recorded	not recorded
RA6	ST239	Argentina	Skin	1996
RA7	ST239	Argentina	Wound	1996
TUR1	ST239	Turkey	not recorded	1996
TUR27	ST239	Turkey	not recorded	1996
TUR9	ST239	Turkey	not recorded	1995
TW20	ST239	UK	not recorded	2003
UCO159	ST239	Argentina	not recorded	not recorded
UK102	ST239	UK	not recorded	1996
UK105	ST239	UK	not recorded	not recorded
URU110	ST239	Uruguay	not recorded	1998
URU34	ST239	Uruguay	not recorded	1997

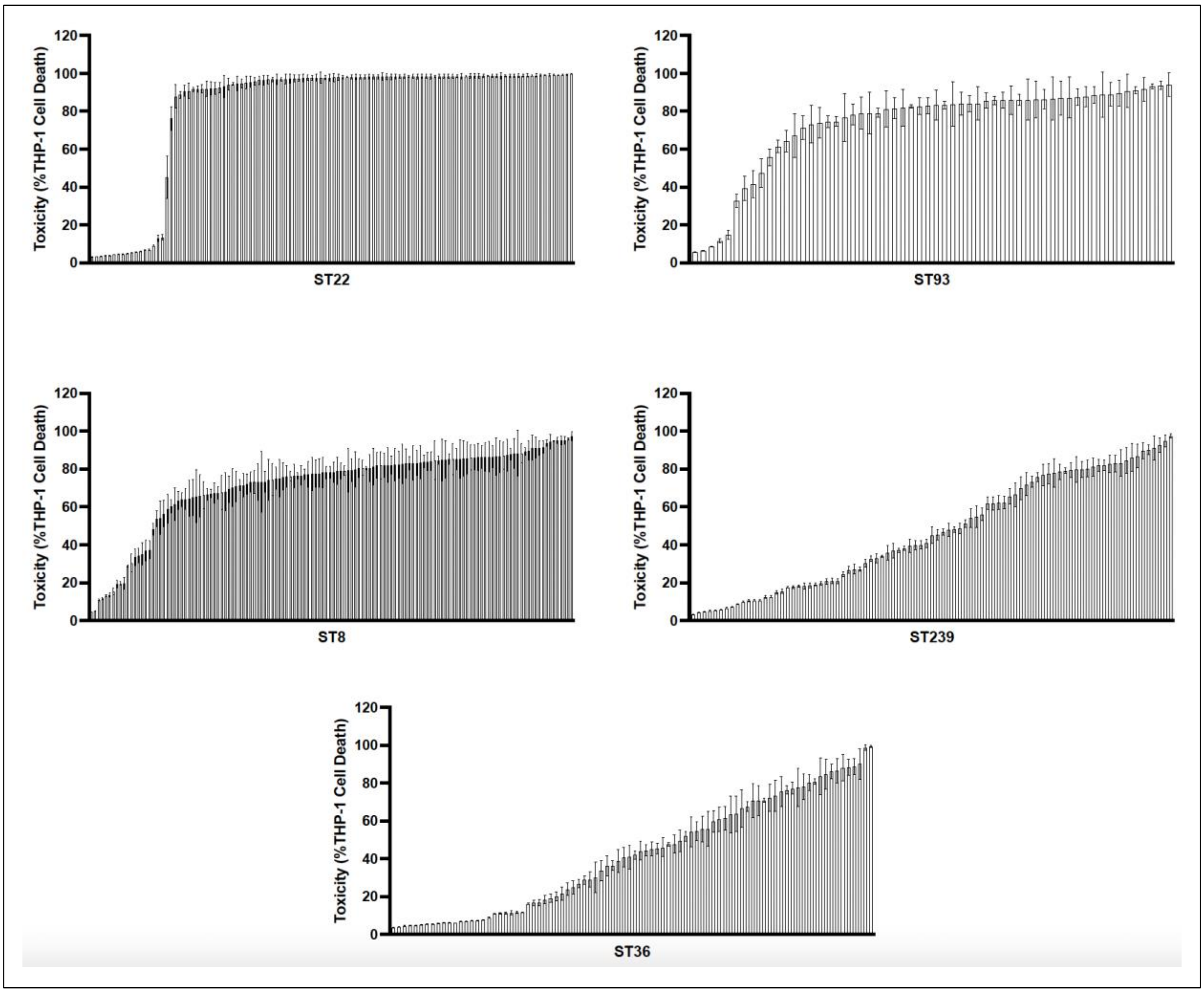

Supplementary Figure 1: Cytotoxicity of 475 MRSA clinical isolates

The cytotoxicity of each clinical isolate from five MRSA multi-locus sequence types (STs) (ST22 (n=110), ST93 (n=58), ST8 (n=134), ST239 (n=87) and ST36 (n=86)) was examined by incubating culture supernatant (either diluted to 30% using sterile TS broth (ST22, ST93 and ST8) or used undiluted (100%; ST239 and ST36)) with cultured THP-1 cells and toxicity as a measure of THP-1 cell death determined using flow cytometry. Each bar represents the mean of one clinical isolate quantified using three biological repeats with error bars representing the standard deviation.

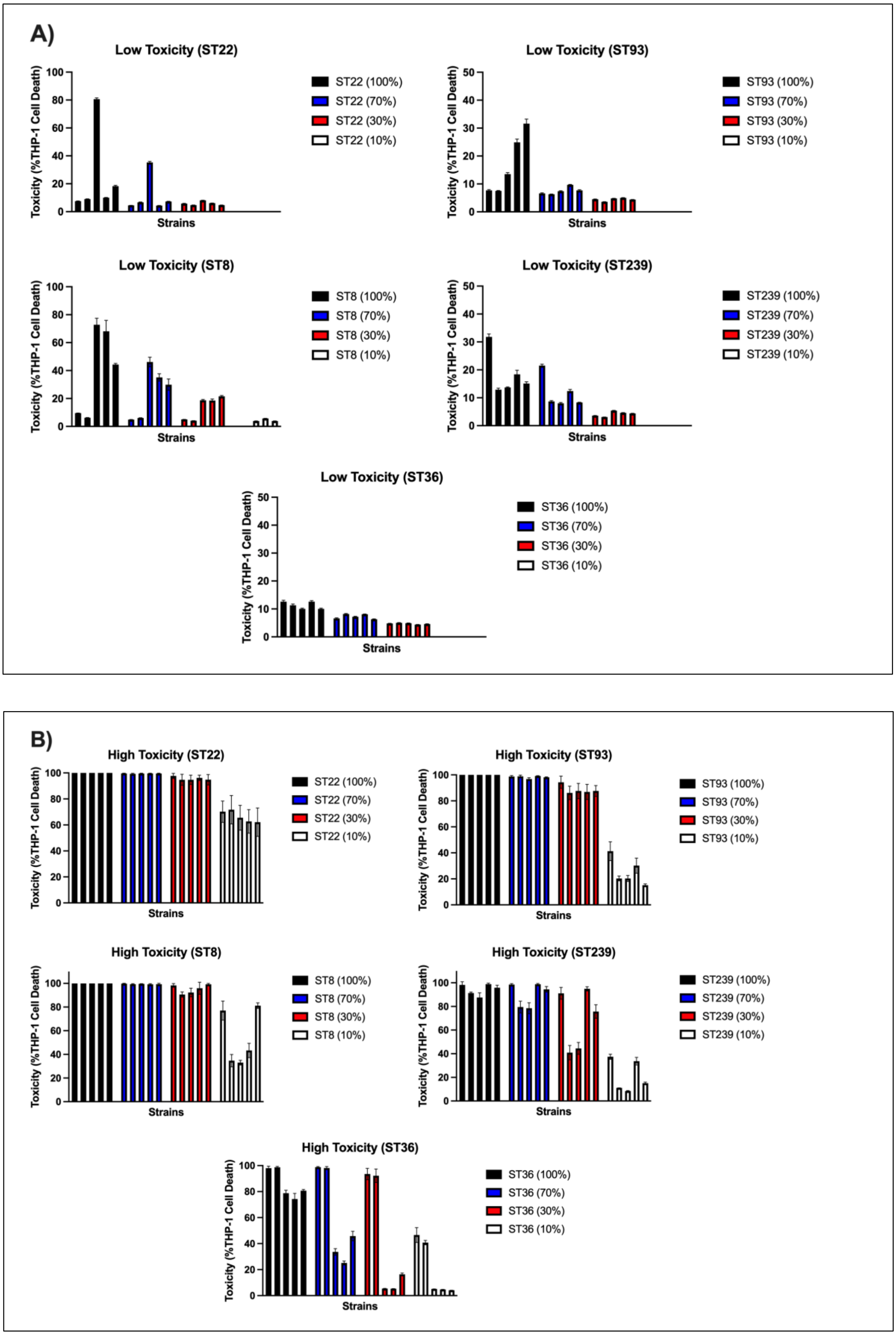
A)
Low Toxicity (ST22)
Toxicity (%THP-1 Cell Death)
Strains
ST22 (100%)
ST22 (70%)
ST22 (30%)
ST22 (10%)
Low Toxicity (ST93)
Toxicity (%THP-1 Cell Death)
Strains
ST93 (100%)
ST93 (70%)
ST93 (30%)
ST93 (10%)
Low Toxicity (ST8)
Toxicity (%THP-1 Cell Death)
Strains
ST8 (100%)
ST8 (70%)
ST8 (30%)
ST8 (10%)
Low Toxicity (ST239)
Toxicity (%THP-1 Cell Death)
Strains
ST239 (100%)
ST239 (70%)
ST239 (30%)
ST239 (10%)
Low Toxicity (ST36)
Toxicity (%THP-1 Cell Death)
Strains
ST36 (100%)
ST36 (70%)
ST36 (30%)
ST36 (10%)
B)
High Toxicity (ST22)
Toxicity (%THP-1 Cell Death)
Strains
ST22 (100%)
ST22 (70%)
ST22 (30%)
ST22 (10%)
High Toxicity (ST93)
Toxicity (%THP-1 Cell Death)
Strains
ST93 (100%)
ST93 (70%)
ST93 (30%)
ST93 (10%)
High Toxicity (ST8)
Toxicity (%THP-1 Cell Death)
Strains
ST8 (100%)
ST8 (70%)
ST8 (30%)
ST8 (10%)
High Toxicity (ST239)
Toxicity (%THP-1 Cell Death)
Strains
ST239 (100%)
ST239 (70%)
ST239 (30%)
ST239 (10%)
High Toxicity (ST36)
Toxicity (%THP-1 Cell Death)
Strains
ST36 (100%)
ST36 (70%)
ST36 (30%)
ST36 (10%)

Supplementary Figure 2: Supernatant dilutions of the lowest and highest toxic isolates from each of the five MRSA sequence types.

Cell-free culture supernatant of the five lowest **(A)** and highest **(B)** toxic isolates from each of the five MRSA STs were diluted to either 10%, 30% or 70% supernatant using sterile TS broth or used undiluted (100%), and toxicity determined as a measure of THP-1 cell death. The cytotoxicity of each isolate was quantified using three biological repeats with each bar representing the mean of individual isolates and error bars indicating the standard deviation.
